# Modulation of radiation-induced oral mucositis (mouse) by dermatan sulfate: effects on differentiation processes

**DOI:** 10.1007/s00066-019-01532-8

**Published:** 2019-11-08

**Authors:** Nilsu Cini, Sylvia Gruber, Zumre Arican Alicikus, Wolfgang Dörr

**Affiliations:** 1 Department of Radiation Oncology, Kartal Dr. Lutfi Kirdar Training and Research Hospital, Istanbul, Semsi Denizer Cad. E-5 Karayolu Cevizli Mevkii, 34890 Kartal, Istanbul, Turkey; 2 Department Radiation Oncology/CD Lab. Med. Radiation Research for Radiation Oncology, Applied and Translational Radiobiology, Medical University/AKH Vienna, Währinger Gürtel 18–20, 1090 Vienna, Austria; 3 Department of Radiation Oncology, Faculty of Medicine, Dokuz Eylul University, Inciralti, 35340 Izmir, Turkey

**Keywords:** Fractionation, Proliferation, Differentiation, Cellular junctions, Mechanical strength

## Abstract

**Purpose:**

During head and neck cancer radiotherapy, oral mucositis is the most frequent early side effect. Systemic dermatan sulfate (DS) administration has been shown to significantly decrease oral mucosal radiation reactions during daily fractionated irradiation (IR) in an established mouse model. The aim of this study was to investigate the mechanism of the oral epithelial differentiation process, during IR alone and in combination with DS treatment in the same mouse model.

**Methods:**

Fractionated IR 5 × 3 Gy/week was given to the snouts of mice over two weeks, either alone (IR) or in combination with daily DS treatment of 4 mg/kg (IR + DS). Groups of mice (*n* = 3) were sacrificed every second day over the course of 14 days in both experimental arms. Their tongue was excised and subjected to immunohistochemical processing.

**Results:**

In the p16 analysis as a proliferation marker, the difference between IR alone and IR + DS in the germinal (proliferation) layer was not significant, not stimulating the proliferation process. For the p21 analysis as a differentiation marker on the functional (differentiation) layer, the difference between IR alone and IR + DS arms was significant, indicating that DS inhibited the differentiation process. In the cytokeratin (CK) analysis as the indicator of cellular skeletal integrity, the percentage of antibody-positive cells was above the normal level in both experimental arms and significantly superior in the IR + DS arm.

**Conclusion:**

The mucosal protective activity of DS, instead of stimulating proliferation, is based on prevention of cell loss by a combination of effects leading to the inhibition of cellular differentiation and an increase in the expression of epithelial mechanical strength between intercellular mechanical junctions.

## Introduction

Oral mucositis is a frequent, often dose-limiting early adverse effect of head and neck cancer radio(chemo)therapy [1, 2], especially when the oral cavity and major salivary glands are in the radiotherapy treatment field [3]. The epithelium of the oral cavity is well suited for its many unique functions, having regional differences in the keratinization of the mucosa [4]. Aside from differences in keratinization, there are regional differences in tissue thickness and cell turnover time. Epithelial homeostasis requires that the rate of cell production equals the rate of desquamation. This concept is known as turnover time [4]. Turnover time also dictates the rate of healing. The balance between epithelial cell production and desquamation has important implications for mucosal health and disease [5]. Proliferation is based on tissue-specific stem cells, which represent a fraction (of unknown size) of the proliferating cells. Stem cells physiologically divide into one new stem cell (a self-renewing system) and one non-stem daughter cell. The latter can, as a transit cell, undergo a limited number of cell divisions before terminal differentiation and loss at the surface [6]. The cell production, either from stem cell proliferation or transit cell division, is limited to the basal layer and the deeper parts of the spinos layer; these are depicted as a germinal layer (stratum germinativum). The functional epithelial layer contains the residual part of spinos layer and the granular layer, which is characterized by basophilic keratohyalin containing granules [7]. During differentiation, cells increase in size and flatten towards the superficial layer. A finely regulated differentiation program process is characterized by the sequential expression of different proteins, coincident with the phenotypic evolution from basal cell to the mature, nonviable stratum layer [8]. The superficial layer is composed of the keratin layer where the keratohyalin is converted in keratin and displays final differentiation with complete keratinization [9, 10]. Murine oral mucosa presents as a multilayered squamous epithelium composed of a germinal layer, a functional layer, and a superficial layer, and is largely comparable to human oral mucosa [11]. Taken together, the oral mucosa represents a perfectly suited model for studying proliferation and differentiation. However, the molecular mechanisms governing mucosal differentiation are still largely unknown [8]. Typical early IR effects are found in turnover tissues, where physiologically permanent cell loss from the differentiated, post-mitotic compartments of the tissue is well balanced by proliferation in the germinal parts of the tissue [12, 13]. Following mechanical or chemical interactions, superficial cell loss triggers cell production in the germinal layer [14]. As a consequence of the proliferative impairment, the reduced cellular supply to the differentiated tissue layers results in progressive hypoplasia and, eventually, incomplete cell depletion [15]. The molecular pathogenesis of this ulcerative epithelial process is still unclear, but the involvement of changes in epithelial differentiation processes is highly likely. DS plays an important role in wound healing, like other glycosaminoglycans (GAG), and it binds to fibroblast growth factor (FGF)-2, which stimulates cell proliferation in response to injury [16]. FGF‑2 has been reported to be connected to DS and activated by DS [17], and it functions as a mitogen that signals mesenchymal cell migration, proliferation, and differentiation [5]. Furthermore, the underlying mechanisms of DS’ mucositis-ameliorating action may include modulation of epithelial differentiation, which needs to be defined [16]. The aim of the present study, therefore, is to investigate and characterize oral epithelial differentiation process during daily fractionated IR alone and in combination with DS treatment in the same well-established mouse model.

## Methods

All underlying in vivo experiments were carried out according to the current animal welfare legislation with the approval of the respective authorities (approval no. BMWF66.009/0039-II/3b/2014).

### Animals and housing

For all experiments, mice of the inbred C3H/Neu strain from the Department for Biomedical Research of the Medical University of Vienna were used to acquire the tissue samples used in the present investigation. No gender-related differences have been found in the radiation response of the oral mucosa [18]. Therefore, both male and female mice were included in this study. Mice were housed in a conventional environment with controlled temperature (22 ± 2 °C) and humidity (55 ± 10%) and a day/night rhythm of 12/12 h. The animals were housed in Makrolon® cages, 1284L Eurostandard type IIL, with a floor area of 530 cm^2^ (Techniplast GmbH, Hohenpeißenberg, Germany), maximum 5 animals per cage, on aspen wood bedding (ABEDD-LAB & VET-Service GmbH, Vienna, Austria) and had free access to standard diet (sniff-Spezialdiäten-GmbH, Soest, Germany) and freshwater ad libitum from standard Perspex drinking bottles. The age of the mice at the onset of the experiments ranged from 8 to 12 weeks [11].

### Irradiation technique and dermatan sulfate treatment

**Fig. 1:**
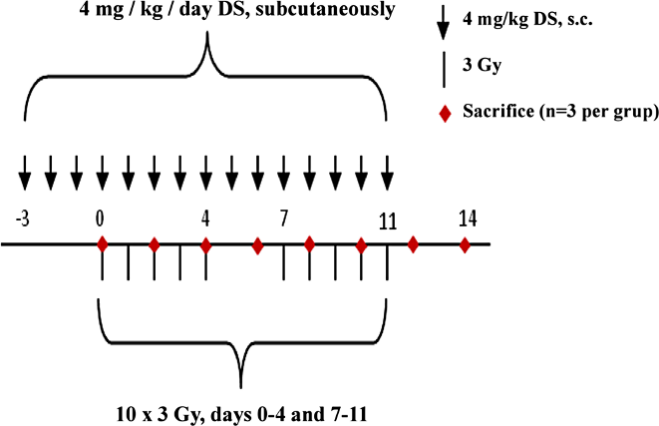
Experimental design. IR comprised fractionated IR with 3 Gy per day over two weeks. No IR was given at the weekend (days 5–6 and 12–13). IR was applied either alone or in combination with daily 4 mg/kg DS treatment over 14 days. At two-day intervals, 3 animals were sacrificed per experimental group, 3 untreated mice (day 0) served as controls

The study comprises two experimental arms: daily fractionated IR alone and in combination with daily systemic administration of DS (IR + DS) arm. DS was administered to the IR + DS group over two weeks, from day 3 to 11, IR started at day 0. In two-day intervals, groups of animals (*n* = 3) were immobilized with 60 mg/kg pentobarbitone sodium, injected intraperitoneally, and were sacrificed by neck dislocation. The experimental design is represented in Fig. 1.

### Irradiation

Snout IR with a dose of 3 Gy/day, 5 fractions/week over 2 weeks, was performed without anesthesia. IR was performed with an X‑ray device (YXLON‑Y.TU320-D03, YXLON-International GmbH, Hamburg, Germany), with a voltage of 200 kV, a tube current of 20 mA, a focus size of 5.5 mm, and a beam filter of 3 mm beryllium (Be) and 3 mm aluminum (Al). The dose rate at the focus–object distance of 45.5 cm was 1027 Gy/min [19]. The unanesthetized animals were guided into plastic tubes (inner diameter 2 cm) and their snouts were positioned in the conical hole (10 mm → 6 pm) in a Perspex block, which closed the front end of the tubes. The rear ends were closed to prevent withdrawal of the mice. Five to eight animals were irradiated simultaneously; dose homogeneity between the individual snout positions was 3.2 ± 0.5%. The beam direction was vertical. The set-up for simultaneous IR of animals was positioned in a standardized way in the central beam of the IR device. An additional 4 mm Al and 0.6 mm Cu beam filter was used. The bodies of the animals were shielded by a 12 mm thick collimator plate consisting of lead equivalent MCP-96 (HEK-Medizintechnik, Lübeck, Germany). The treatment field encompassed the area from the eyes to the throat, thus including the entire tongue. The mice were irradiated between 10:00 am and 1:00 pm [20].

### Dermatan sulfate administration

A daily dose of 4 mg/kg DS (Sigma-Aldrich, MO, USA, Cat.no. C3788) dissolved in saline at a concentration of 1 mg/ml was administered subcutaneously over varying time intervals. On IR days, the drug was administered two hours after IR.

### Immunohistochemical staining

Immunohistochemical staining performed by Leica Biosystems BOND RX for formalin-fixed, paraffin-embedded and cut in 3 μm thick tongue sections according to optimized (for the specific tissue) standard protocols. Histological preparation procedures have been reported in detail previously [21]. Stainings comprised 30 min incubation at 95 °C, dewaxing with Leica Bond Dewax solution (Leica-Biosystems, Inc., Buffoalo. Groove, IL; Cat.no. AR9222), antigen retrieval with Bond Epitope Retrieval‑1 solution (Leica Biosystems, Inc., Buffoalo. Groove, IL; Cat. no. AR9961), and blocking of unspecific binding sites with 2% goat serum. Primary antibody binding was visualized with diaminobenzidine chromogen and a hematoxylin counterstain, using the Leica Bond Refine Detection kit (Leica-Biosystems, Inc., Buffalo. Groove, IL; Cat. no. DS9800). Primary antibodies were diluted in the Leica Bond Antibody Diluent buffer (Leica-Biosystems, Inc., Buffoalo. Groove, IL; USA, Cat. no. AR9352) as follows [20]; anti-p16 ARC antibody 1:50 (Abcam, Cambridge, MA, USA; Cat.no. 51243; rabbit-monoclonal), anti-p21 Ras antibody 1:100 (Abcam, Cambridge, MA, USA; Cat.no. 86696; mouse-monoclonal), and anti-cytokeratin 5 antibody 1:5000 (Abcam, Cambridge, MA, USA; Cat.no. 52635; rabbit monoclonal).

### Evaluation of stained cells

Microscopic analyses were performed field by field with an Axio Lab. A1-HAL. 35 (Carl-Zeiss Microscopy, LLC, Thornwood, NY, USA) at 400 × 0.65 magnification by using a square grid. The lower mouse tongue epithelium was analyzed from the tip (field 1) to the end of the tongue. An average of five visual fields were evaluated (from 3 to 8). In each visual field, the number of marker-positive cells was normalized to the total number of cells. The fraction of positive cells was evaluated separately for the germinal and the functional epithelial layer. Additionally, the staining intensity, corresponding to the amount of protein expressed, was assessed semi-quantitatively with an arbitrary score from 0 (no signal), 1 (weak signal), 2 (intermediate signal), to 3 (strong signal). Staining intensity was scored per visual field, not for each marker-positive cell individually. Also, epithelial thickness was measured for the germinal, functional, and keratin layer of the epithelium at two representative positions of the ventral surface of the tongue. Marker-positive cells and their respective staining intensity were evaluated by two independent and experienced researchers in a blinded fashion after extensive training. Intra-observer variability was found to be negligible [20].

### Statistics

For statistical analysis, the SPSS 17 statistical software (SPSS Inc., Chicago, IL, USA), for graphical representation, GraphPad Prism 5 (GraphPad Software, Inc., CA, USA) was used. Mean values and standard deviation (SD) were calculated for each experimental group. The analysis of variance (one-way ANOVA [Analyis of variance]) was used to test for the significance of a difference between the mean values of experimental arms. A *p*-value of <0.05 was regarded as statistically significant [20].

## Results

**Fig. 2:**
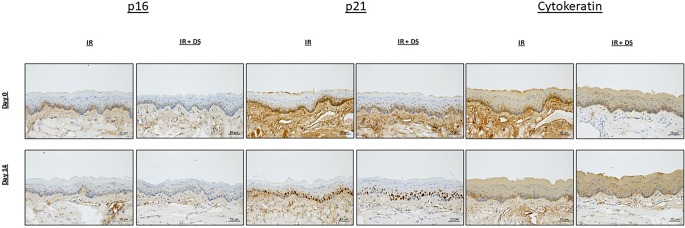
p16, p21, and CK expression during fractionated IR ± DS. Representative histophotographs of p16-, p21-, and CK-stained lower mouse tongue epithelia were taken on day 0 (unirradiated and untreated controls) and day 14. Scale bar: 50 µm

Representative histophotographs of immunohistochemical staining for p16, p21, and CK in untreated control mucosa on day 0 and on day 14 are presented in Fig. 2.

### p16 labeling index and staining intensity

#### Germinal layer

**Fig. 3:**
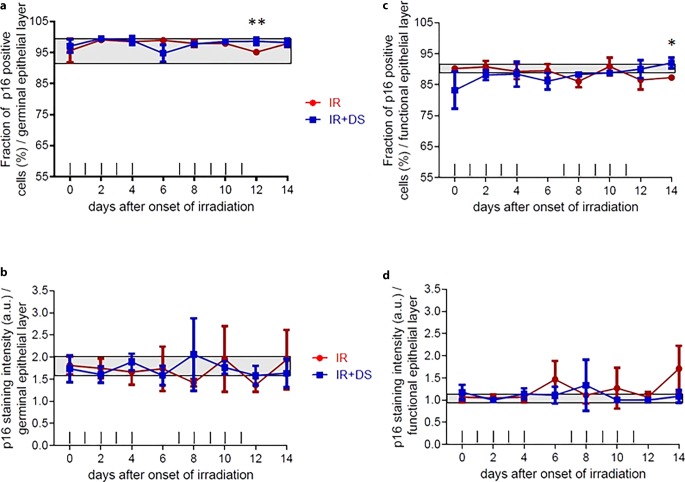
Effect of fractionated IR ± DS number of cells immunohistochemically positive stained cell percentage and staining intensity for p16. p16 expression changes were analyzed in the germinal and the functional epithelial compartment. The staining signal intensity was scored semi-quantitatively with an arbitrary score of 0 (no signal), 1 (weak), 2 (intermediate), or a maximum of 3 (strong). p16 was analyzed in 3 specimens per experimental arm, every second day over the course of 14 days. *Data points* represent the mean of 3 animals, *error bars* indicate ±1 SEM. The *shaded areas* illustrate the mean (±1 SEM) from 3 control animals. The fractionation protocol is indicated on top of the abscissae. *Asteriskp* < 0.05; *doubleAsteriskp* < 0.01

The percentage of p16-positive cells in the control group was 91.2–98.2% in both experimental arms. Significance between experimental armsIR-only and IR+DS was only observed at day 12 *p* = 0.009 (Fig. 3a). The staining intensities deviated from the control group interval, which is 1.6–2.0 arbitrary unit (a.u.) for the IR-only arm on the 6th, 10th, and 14th day; and for the IR + DS arm on the 8th and 14th day, and no significant difference between two experimental arms (Fig. 3b).

#### Functional layer

The percentage of p16-positive stained cells did not represent a significant difference between IR-only and IR + DS arms, except on day 14, *p* = 0.02, and control group range 89.5–91.2% (Fig. 3c). The staining intensity was increased in the IR-only arm at days 6, 10, and 14 from the control group range which was between 1–1.2 a.u., no significant difference between two experimental arms (Fig. 3d).

### p21 labeling index and staining intensity

#### Germinal layer

**Fig. 4:**
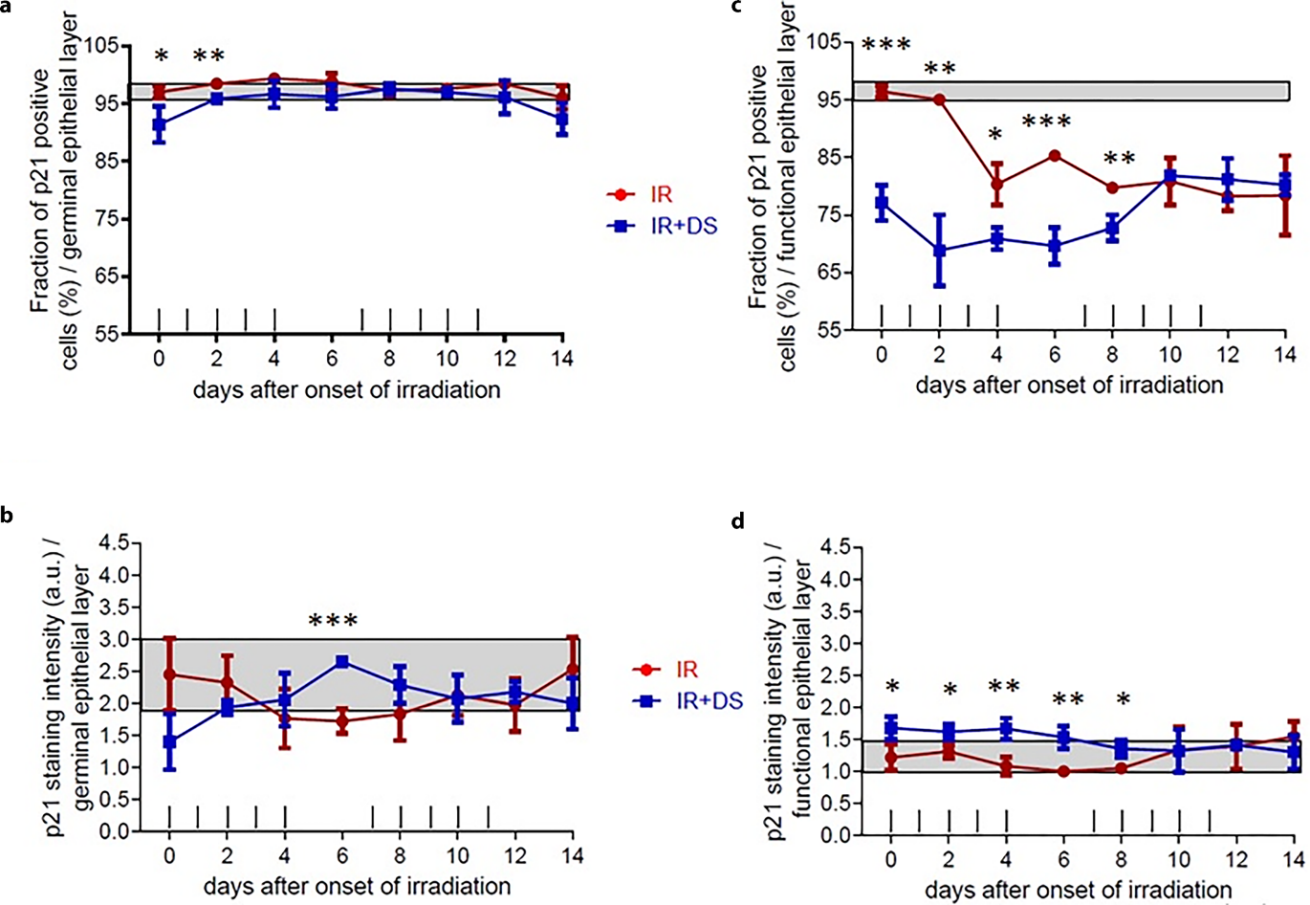
Effect of fractionated IR ± DS number of cells immunohistochemically positive stained cell percentage and staining intensity for p21. p21 expression changes were analyzed in the germinal and the functional epithelial compartment. The staining signal intensity was scored semi-quantitatively with an arbitrary score of 0 (no signal), 1 (weak), 2 (intermediate), or a maximum of 3 (strong). p21 was analyzed in 3 specimens per experimental arm, every second day over the course of 14 days. *Data points* represent the mean of 3 animals, *error bars* indicate ±1 SEM. The *shaded areas* illustrate the mean (±1 SEM) from 3 control animals. The fractionation protocol is indicated on top of the abscissae. *Asteriskp* < 0.05; *double Asteriskp* < 0.01; *tripleAsteriskp* < 0.001

The percentage of p21-positive stained cells in the control arm was 95.8–97.8%. The minimum values were as follows: for the IR-only arm 83.2% on day 14 and for the IR + DS arm 89.5% on day 0 and day 14. Significance was *p* = 0.04 (day 0), *p* = 0.003 (day 2) (Fig. 4a). The staining intensity significance between IR-only and IR + DS arms only observed at day 6 *p* = 0.001. For IR + DS arm, the lowest level of staining intensity was on day 0, and increased to the control group level (1.8–2.8 a.u.) until day 4 and later on progressed by decreasing until day 14 (Fig. 4b).

#### Functional layer

The percentage of p21-positive stained cells is significantly lower in the IR + DS arm compared to the IR-only arm until day 10. From day 10 onwards, IR + DS arm and IR-only arm reached the close values. The significance between experimental arms was *p* = 0.001 (day 0), *p* = 0.002 (day 2), *p* = 0.016 (day 4), *p* = 0.001 (day 6), *p* = 0.008 (day 8). The control group range was 95.8–97.7% (Fig. 4c). Staining intensity was significantly higher in the IR + DS arm until the 10th day when compared with the IR-only arm. It was observed that the values for the IR-only arm varied within the control group range which was 1–1.40 a.u. The significance between experimental arms was *p* = 0.04 (day 0), *p* = 0.03 (day 2), *p* = 0.01 (day 4), *p* = 0.007 (day 6), *p* = 0.03 (day 8) (Fig. 4d).

### Cytokeratin labeling index and staining intensity

#### Germinal layer

**Fig. 5:**
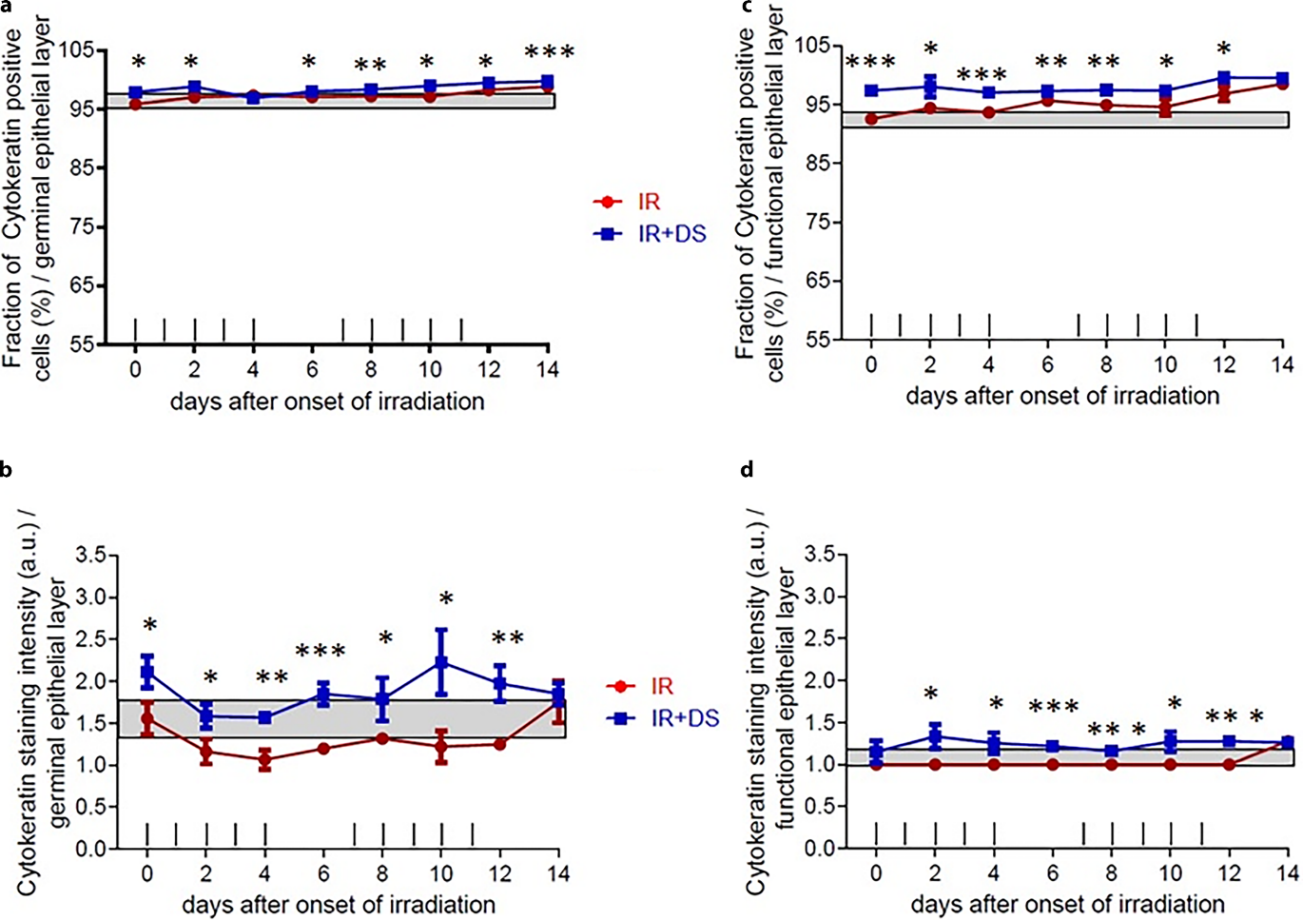
Effect of fractionated IR ± DS number of cells immunohistochemically positive stained cell percentage and staining intensity for CK. CK expression changes were analyzed in the germinal and the functional epithelial compartment. The staining signal intensity was scored semi-quantitatively with an arbitrary score of 0 (no signal), 1 (weak), 2 (intermediate), or a maximum of 3 (strong). CK was analyzed in 3 specimens per experimental arm, every second day over the course of 14 days. *Data points* represent the mean of 3 animals, *error bars* indicate ±1 SEM. The *shaded areas* illustrate the mean (±1 SEM) from 3 control animals. The fractionation protocol is indicated on top of the abscissae. *Asteriskp* < 0.05; *double Asteriskp* < 0.01; *tripleAsteriskp* < 0.001

The percentage of CK-positive cells did not fall below the control group interval (95.2–96.3%) in either arm. The IR + DS arm was significantly higher than the IR-only arm, except on day 4. The significance between experimental arms was *p* = 0.011 (day 0), *p* = 0.015 (day 2), *p* = 0.034 (day 6), *p* = 0.007 (day 8), *p* = 0.021 (day 10), *p* = 0.02 (day 12), and *p* = 0.00 (day 14; Fig. 5a). The staining intensity was significantly higher in the DS-applied arm, *p* = 0.025 (day 0), *p* = 0.025 (day 2), *p* = 0.003 (day 4), *p* = 0.001 (day 6), *p* = 0.035 (day 8), *p* = 0.015 (day 10), *p* = 0.004 (day 12). Control group range 1.67–1.33 a.u. (Fig. 5b).

#### Functional layer

The percentage of CK-positive cells was near the upper limit of the control group in the IR-only arm, while the IR + DS arm did not fall below the control group range (91.9–92.9%). Significantly higher CK-positive percentage was observed until day 14 on IR + DS arm, *p* = 0.001 (day 0), *p* = 0.028 (day 2), *p* = 0.001 (day 4), *p* = 0.002 (day 6), *p* = 0.003 (day 8), *p* = 0.04 (day 10), *p* = 0.022 (day 12; Fig. 5c). Also, the staining intensity was significantly higher in the DS-applied arm and IR only arm observed around the control group range which was 1 a.u. The significance between experimental arms was *p* = 0.016 (day 2), *p* = 0.024 (day 4), *p* = 0.000 (day 6), *p* = 0.000 (day 8), *p* = 0.015 (day 10), *p* = 0.000 (day 12; Fig. 5d).

### Epithelial thickness and cell numbers

#### Epithelial thickness

**Fig. 6:**
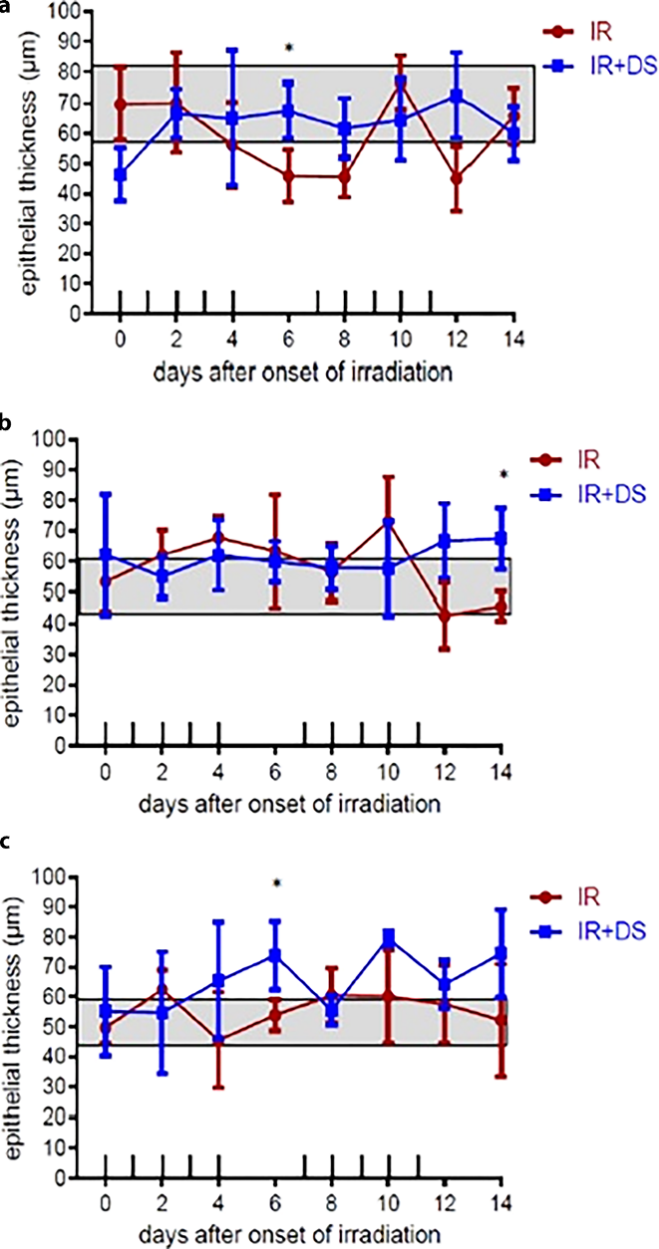
Relative epithelial thickness during daily fractionated IR ± DS treatment on the epithelial expression of p16, p21, and CK. Epithelial thickness was determined at representative spots in each tongue section. p16, p21, and CK were analyzed in 3 specimens per experimental arm, every second day over the course of 14 days. *Data points* represent the mean of 3 animals, *error bars* indicate ±1 SEM. The *shaded areas* illustrate the mean (±1 SEM) from 3 control animals. The fractionation protocol is indicated on top of the abscissae. *Asteriskp* < 0.05; *doubleAsteriskp* < 0.01; *tripleAsteriskp* < 0.001

The average epithelial thicknesses for the IR-only arm were 57.5 µm; 3.59 µm (p16); 57.9 µm (p21), and 55.2 µm (CK); for the IR + DS arm 63.15 µm; 3.63 µm (p16); 61 µm (p21), and 65.33 µm (CK). Significance between experimental arms for p16 was *p* = 0.04 (day 6; Fig. 6a), for p21 *p* = 0.03 (day 14; Fig. 6b), and for CK *p* = 0.05 (day 6; Fig. 6c). DS-applied arm average epithelial thicknesses were higher than the IR-only arm for p16, p21, and CK.

### Cell numbers

#### Total

**Fig. 7:**
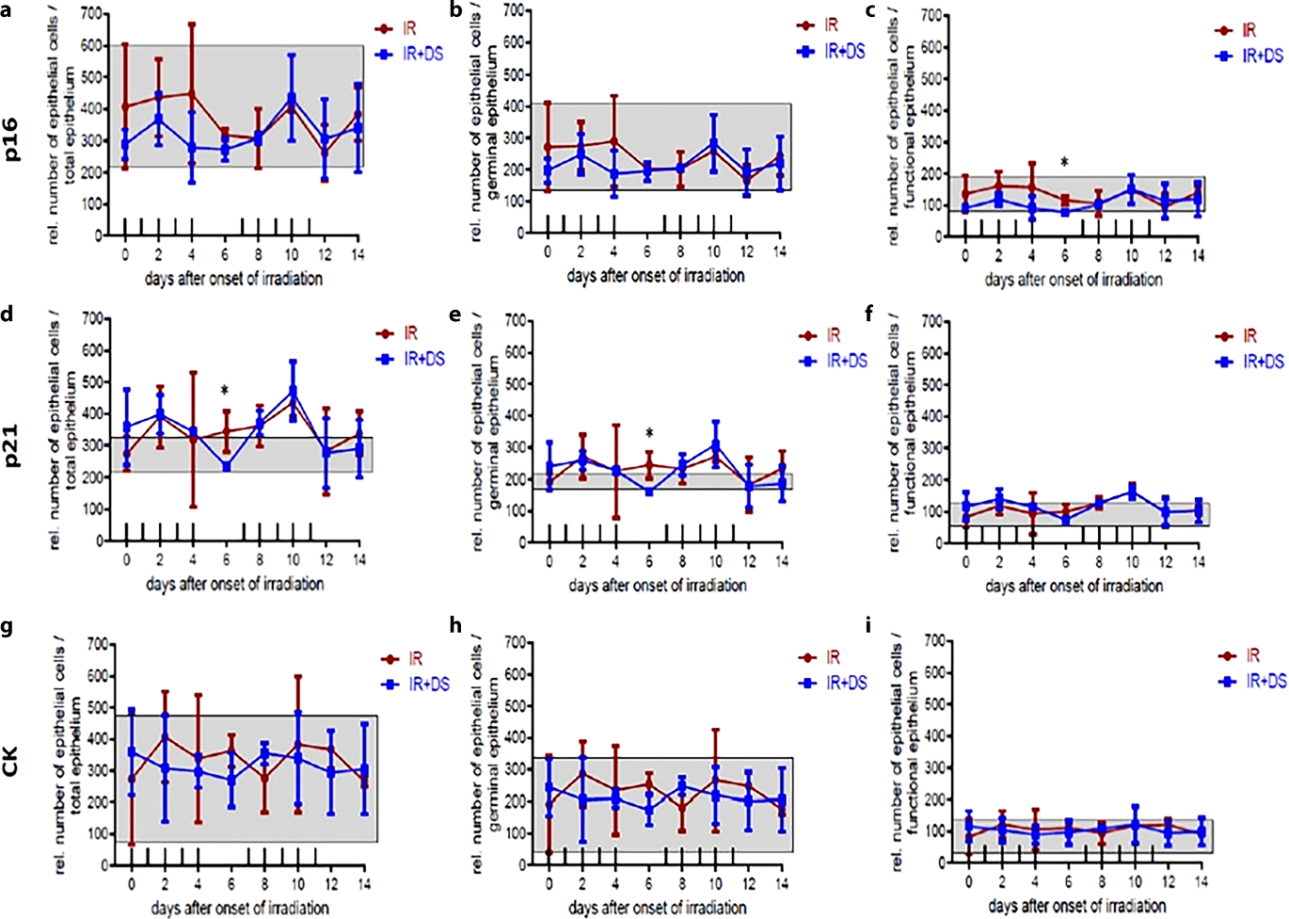
Relative number of epithelial cells during daily fractionated IR ± DS treatment on the epithelial expression of p16, p21, and CK for total epithelium, germinal epithelium, and functional epithelium. Cell numbers were determined at representative spots in each tongue section. p16, p21, and CK were analyzed in 3 specimens per experimental arm, every second day over the course of 14 days. *Data points* represent the mean of 3 animals, *error bars* indicate ±1 SEM. The *shaded areas* illustrate the mean (±1 SEM) from 3 control animals. The fractionation protocol is indicated on top of the abscissae. *Asteriskp* < 0.05; *doubleAsteriskp* < 0.01; *tripleAsteriskp* < 0.001

The average total cell numbers for the IR-only arm were 349,371 for p16, 343 for p21, and 335 for CK; for the IR + DS arm 328,325 (p16), 343 (p21), and 316 (CK). Significance between experimental arms was only given for p21 *p* = 0.043 (day 6; Fig. 7d).

#### Germinal epithelium

The average number of cells in the IR-only arm was 233,239 (p16), 232 (p21), and 230 (CK); for IR + DS arm 218,217 (p16), 226 (p21), and 213 (CK). Significance between experimental arms was only given for p21 *p* = 0.028 (day 6; Fig. 7e).

#### Functional epithelium

The average number of cells in the IR-only arm was 116,132 (p16), 112 (p21), and 105 (CK); for the IR + DS arm 110,108 (p16), 117 (p21), and 103 (CK). Significance between experimental arms was only given for p16 *p* = 0.012 (day 6; Fig. 7c). There is no strong significance between IR-only and IR + DS arms for total, germinal, and functional cell numbers.

## Discussion

Oral mucositis refers to erythematous and painful ulcerative lesions of the oral mucosa observed in patients with head and neck cancer who are treated with chemo- and/or radiotherapy [22]. DS has been shown to significantly increase oral mucosal radiation tolerance during daily fractionated IR in an established mouse model [23]. One of the basis mucosal protective property of DS anticoagulant activity, which increases blood support in areas where vascular structures are narrowed, is thought to reduce inflammation [17]. Gruber et al. found out the advantage and effect of daily fractionated radiotherapy on adherent vs. tight junctions in the recovery of oral mucositis [20]. As a result, the effect of IR on the epithelial cell junctions is significantly superior, and reduces the probability of development of oral mucositis. Also, Gruber et al. observed the effect of DS on the healing of oral mucositis, through the epithelial junction, hypoxia, and inflammation markers. The development of epithelial integrity and cohesion caused by DS prevents the loss of quality cells [24]. Our study focused to observe effects of DS on radiation-induced oral mucositis and due to this aim immunohistochemical staining planned on cell proliferation marker (p16), cell differentiation marker (p21), and indicative of cellular skeletal integrity antibody (CK). The activity of p16 in the phases of the cell cycle has been investigated, and it has been associated with the S‑phase which indicates proliferation [25]. In our study, p16 did not make a significant difference in the germinal (proliferation) layer when the experimental arms compared. This supports the knowledge from the Hertzendorfer et al. study that DS did not stimulate the proliferation process [19]. p21 is involved in the process of terminal differentiation [26] and inhibits the activity of cyclin-dependent kinase which controls the transition from G1 to S‑phase during the cell cycle [27]. In our study, the percentage of p21-positive cells decreased for the functional (differentiation) layer in the DS-applied arm. The oral mucosal protective feature of DS is considered to inhibit the differentiation process by stimulating the junction. In this case, cells whose cellular differentiation is inhibited cannot escape from the layer in which they are located to the surface, and the number of cells before IR will be prevented by this effect. Our study is one of the first to examine the epithelial differentiation process. The main function of CK is to give mechanical strength to the epithelial cells [28]. Radiation induces damage to the epithelium, with the release of CK resulting in cell death and inhibition of basal cell proliferation [29–31]. These epithelial changes possibly lead to differential expression of CK in each phase [32, 33]. Keratins are the predominant cytoskeletal protein of stratified keratinized epithelial cells and are the most sensitive markers of epithelial differentiation and proliferation [34–36], because their expression is both region specific and differentiation specific [37]. However, no previous study has focused on CK expression in oral radiation-induced mucositis. Bonan et al. observed that increased CK expression can be associated with the reactive proliferation of the epithelium and increasing resistance of the oral mucosa during the initial phases of IR [38]. The results of our study for the percentage of CK-positive stained cells, the DS-applied arm superior to IR-only arm in both germinal and functional layers, which shows that DS supports mechanical strength at the cellular level. Besides, when the significance between germinal and functional layers is compared for each experimental arm, the effect of DS on the mechanism of inhibition of differentiation made a significant difference in the functional (differentiation) layer in favor of the IR + DS arm. But still, the percentage of positive cells did not fall below the control group range of percentage of positive cells for either experimental arm. This effect supports the positive activity of fractionated IR on junctions [20]. In this sense, our study is one of the leading studies examining the epithelial differentiation process of CK expression on radiation-induced oral mucositis.

Kozma et al. investigated the effect of DS on breast cancer tumor cell samples instead of normal tissues and showed that DS often causes adverse effects on breast cancer cells, and high doses of DS lead to a decrease in breast cancer cell proliferation. The results of this study clearly demonstrated the complex role of DS in the cancer setting and revealed the anti-cancer potential of GAG [39].

## Conclusion

Dermatan sulfate inhibits differentiation by stimulating the junctions and supports mechanical strength at the cellular level and creates the integrity of the epithelial layer, while does not stimulate the proliferation. Based on these curative effects of DS, protection of existing cells will reduce the possibility of clinical manifestation, duration, and/or severity of oral mucositis. Our study is one of the first studies examining the epithelial differentiation process on radiation-induced oral mucositis by differentiation marker p21 and cytokeratin expression. As a result in our study, we observed the normal tissue-protective effects of DS on the oral mucosa and Kozma et al. reported the anti-cancer potential of DS on breast cancer samples.

The promising features of DS including the mechanisms of the mucosa-protective activity, anti-cancer potential, inhibition of differentiation and not effecting the proliferation could be examined on the head and neck tumour sections, via proliferation and differentiation markers for further research.
